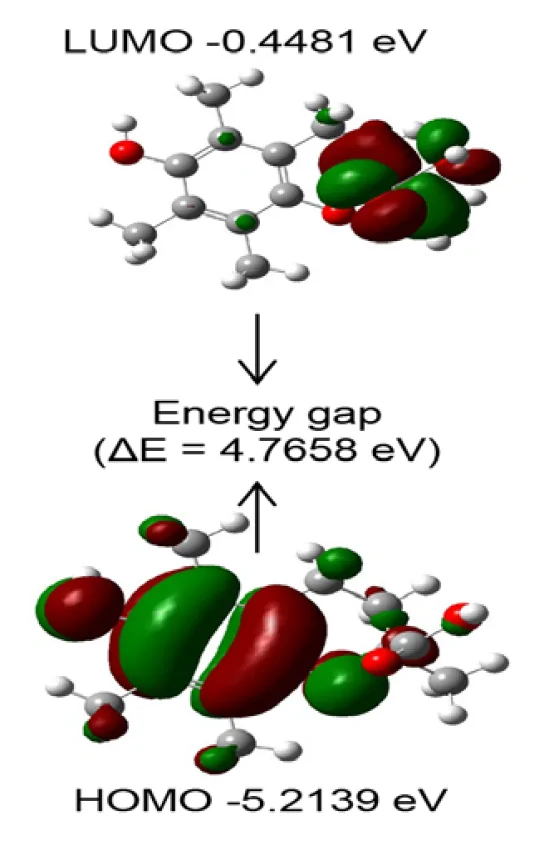# Antioxidant Metabolites from *Justicia gendarussa* Fractions based on UHPLC-Q-Orbitrap-HRMS Metabolomics and Density Functional Theory Approach

**DOI:** 10.21315/tlsr2026.37.3.6

**Published:** 2026-10-15

**Authors:** Faizal Maulana, Waras Nurcholis, Trivadila, Irmanida Batubara

**Affiliations:** 1 Department of Chemistry, Faculty of Mathematics and Natural Sciences, IPB University, Bogor, 16680 West Java, Indonesia; 2 Tropical Biopharmaca Research Center, IPB University. Jl. Taman Kencana No. 3, Bogor, 16128 West Java, Indonesia; 3 Department of Biochemistry, Faculty of Mathematics and Natural Sciences, IPB University, Bogor, 16680 West Java, Indonesia

**Keywords:** DFT, Gendarussa, Heatmap, OPLS-DA, Principal Component Analysis, PCA

## Abstract

The herb *Justicia gendarussa*, known as gendarussa, has the potential as an antioxidant. A previous study has identified that ethanol extract from *J. gendarussa* has the highest antioxidant activity. The study profiled the metabolites of *n*-hexane, ethyl acetate, *n*-butanol and water fractions of its ethanol extract using ultrahigh performance liquid chromatography-high resolution mass spectrometry (UHPLC-Q-Orbitrap HRMS). The antioxidant capacities of metabolites were determined using 2,2-diphenyl-1-picrylhydrazyl (DPPH), 2,2′-azinobis-(3-ethylenebenzothiazoline)-6-sulfonic acid (ABTS), ferric ion reducing antioxidant power (FRAP) and cupric reducing antioxidant capacity (CUPRAC) method combined with chemometric technique. Finally, a computational density functional theory (DFT) was carried out to ensure the ability of the active compounds as an antioxidant. The results showed that the highest antioxidant capacity of the DPPH method was on the *n*-butanol fraction of 40.5 μmol (trolox equivalent) TE/g dry fraction, while the ABTS, FRAP and CUPRAC methods were on the ethyl acetate fraction with antioxidant capacity were 69, 189 and 624 μmol TE/g dry fraction. Principal component analysis (PCA) and heatmap succeeded in classifying the metabolites in *J. gendarussa* fraction based on polarity. There were 37 and 21 identified metabolites in positive and negative ionisation, respectively. Using orthogonal partial least square-discriminant analysis (OPLS-DA), the predictive active antioxidant metabolites were gendarusin A, justridusamide A, sesamol, 6-{[(Benzyloxy)carbonyl]amino}-6-deoxy-1,2-O-isopropylidene-α-D-glucofuranose, methyl 2-[cyclohex-2-en-1-yl(hydroxy) methyl]-3-hydroxy-4-(2-hydroxyethyl)-3-methyl-5-oxoprolinate and 3,4-Dihydroxy-6-(N-ethylamino) benzamide. In addition, 14 unknown metabolites in the ethyl acetate fraction were predicted to be active antioxidants, which can be isolated for further research to obtain new metabolites. Density functional theory (DFT) proves that 6-{[(Benzyloxy)carbonyl]amino}-6-deoxy-1,2-O-isopropylidene-α-D-glucofuranose and gendarusin A have a smaller energy gap than trolox as a standard, indicating it has a better antioxidant ability. Therefore, those two compounds are the best antioxidant compounds. Additional tests *in vitro* or *in vivo* research on the isolation compounds results need to be carried out to prove the research results further.

**HIGHLIGHTS**

- *Justicia gendarussa* demonstrates significant potential as a promising natural source of active antioxidants.
- Metabolomics successfully profiled and identified active compounds with chemometrics, such as PCA, heatmaps and OPLS-DA, classified metabolites by polarity, identifying six active antioxidant compounds and isolable unknowns in the ethyl acetate fraction.
- Density functional theory (DFT) validated the theoretical determination of the most effective antioxidant metabolites within the plant.

## INTRODUCTION

*Justicia gendarussa* (Acanthaceae), commonly known as gendarussa, is a prominent medicinal plant found across several Asian countries, including Indonesia, India, Malaysia and Sri Lanka (Ratih *et al*. 2019). It was selected for this study due to its diverse and unique ethnopharmacological profile; it serves as a traditional male contraceptive in Papua, Indonesia, while in India, it is used to treat conditions including headaches, bronchitis, arthritis, jaundice, otalgia, digestive disorders, fever, cancer and for UV protection (Putri *et al*. 2020). This therapeutic versatility is driven by a rich composition of secondary metabolites, including phenolics, alkaloids, steroids and terpenoids (Widodo *et al*. 2019). Notably, the plant contains specific flavonoids, including gendarusins A–E, with gendarusin A as the major metabolite (Kumar *et al*. 2018; Ratih *et al*. 2019). Other bioactive constituents identified include the alkaloid group brazoides A–D (Souza *et al*. 2017; Zhang *et al*. 2021a), the steroid stigmasterol (Uddin *et al*. 2011), and terpenoids such as (+) and (−)-genpenterpene A (Zhang *et al*. 2021b). These metabolites provide a distinct chemical scaffold for the development of potential antioxidants.

The significance of identifying potent antioxidants lies in their ability to mitigate the damage caused by oxidative stress (Chaudhary *et al*. 2015). Such oxidative damage is fundamentally linked to the progression of various chronic diseases, including neurodegenerative disorders, diabetes mellitus, cardiovascular diseases, rheumatoid arthritis, cataracts, respiratory diseases and various types of cancer (Di Meo & Venditti 2020). These pathologies are driven by reactive oxygen species (ROS) produced by cellular metabolism, including hydrogen peroxide (H_2_O_2_), hydroxyl radicals (OH•), superoxide anion radicals (O_2_^−^•) and peroxyl radicals (ROO•) (Çalişkan & Çalişkan 2021). While previous research has confirmed the antioxidant capacity of *J. gendarussa* leaf ethanol extracts (Kuber 2021) and identified high DPPH scavenging activity in the ethyl acetate fraction of the aerial parts (Zhang *et al*. 2021a), significant knowledge gaps remain. Specifically, no study has yet reported the antioxidant potential across a different polarity like *n*-hexane, ethyl acetate, *n*-butanol and water fractions using an integrated metabolomics and density functional theory approach.

To address these gaps, this study utilises metabolomics combined with orthogonal partial least squares-discriminant analysis (OPLS-DA) to correlate biological activity with chemical profiles. This methodology has identified active markers in other plants, such as rosmarinic acid in *Orthosiphon aristatus* (Maulana *et al*. 2022) and epicatechin/epigallocatechin in *Xylocarpus granatum* (Heryanto *et al*. 2023). Furthermore, we employ computational chemistry through density functional theory (DFT) to validate the molecular reactivity and thermodynamic properties of these compounds (Kaddouri *et al*. 2020). This approach provides a comprehensive understanding of antioxidant mechanisms through gap energy (ΔE) analysis (Ramadhan 2021). By integrating metabolomics and DFT, this research offers a more rapid and cost-effective alternative to traditional bioassay-guided isolation (Maulana *et al*. 2022). Consequently, this study aims to identify and theoretically evaluate the active antioxidant compounds in specific fractions of *J. gendarussa*.

## MATERIALS AND METHODS

### Cultivation and Sample Preparation

Plant cultivation takes place in Biopharmaca Cultivation Conservation Unit, Cikabayan Gardens Block C, IPB Dramaga Bogor Campus, Indonesia, located at 6°3′49″S and 106°42′57″E, at 240 m above sea level. The cultivation for all treatment plants received 100 kg/ha of P_2_O_5_ fertiliser, 150 kg/ha of KCl, 270 kg/ha nitrogen fertiliser and 20 tons/ha of cow manure. The fertiliser is mixed and distributed in polybags (10 cm × 15 cm) around plants grown for one month, then transferred to larger polybags (30 cm × 30 cm). The leaves and stems of *J. gendarussa*, which had been fertilised for four months, were harvested at age of 4 months. In addition, the sample’s dried weight was determined by drying it in an oven at 45°C for 2 × 24 h.

### Preparation of Fractions

Sample of 10 g *J. gendarussa* aerial parts (leaves and stems) were extracted with 100 mL of ethanol p.a. in a ratio of 1:10 for 24 h in a dark place at room temperature. Multilevel fractionation was carried out using *n*-hexane, ethyl acetate, *n-*butanol and water as the solvent with a volume ratio of 1:2 for each solvent. A sample of 1 g of the extract was dissolved in 25 mL of distilled water and then put into a 500 mL separating funnel. The solution was added with 50 mL of solvent. The layer of solvent was separated into bottles. The resulting *n*-hexane, ethyl acetate, *n*-butanol and water fractions were then evaporated using a rotary evaporator. Fractionation with each solvent was repeated three times.

### Antioxidant Capacity

The DPPH method refers to Batubara *et al*. (2020), a 100 μL of the sample solution of *J. gendarussa* fractions and 100 μL of 125 μM DPPH was added to a 96-well clear polystyrene microplate. Mixed solution was incubated for 30 min in a dark place at room temperature. The absorbance was measured with a microplate nano spectrophotometer at 515 nm wavelength.

The ABTS method refers to Nurcholis *et al*. (2022), a 20 μL of the fractions sample and 180 μL of ABTS reagent was added on a 96-well clear polystyrene microplate. Then, the mixed solution was incubated for 6 min. The absorbance of the mixed solution was measured using a microplate nano spectrophotometer at 734 nm wavelength.

The FRAP method refers to Batubara *et al*. (2020), a 10 μL samples of *J. gendarussa* fractions and 145 μL of FRAP was added into a 96-well clear polystyrene microplate and then incubated in a dark place at room temperature for 4 min. The absorbance of the sample was read at 593 nm wavelength using a microplate nano spectrophotometer.

The CUPRAC method refers to Nurcholis *et al*. (2022), a 50 μL of the fractions sample was added to 50 μL of 10^−2^ M CuCl_2_ solution, 50 μL of NH_4_Ac buffer 1 M with pH 7 and 50 μL of 7.5 × 10^−3^ M neocuproin on a microplate. Then, the mixed solution was incubated in a dark room for 30 min at room temperature. Then, the absorbance of the solution was calculated at 450 nm using a microplate nano spectrophotometer. All methods used calibration curves with standard trolox solution with various concentrations. The results are equivalent to μmol trolox equivalent per gram dry weight (μmol TE/g dry fraction weight) (Nurcholis *et al*. 2022).

### Metabolite Identification Using UHPLC-Q-Orbitrap HRMS

Metabolites were identified using the Vanquish UHPLC-Q Exactive Plus Orbitrap-High Resolution Mass Spectrometer. A 5 mg sample was dissolved in 1 mL of LC-MS grade methanol, then sonicated for 10 min and filtered through a 0.2 μm filter membrane. Separation chromatography was performed using an AccucoreTM Vanquish C18+ column (1.5 μm, 120 Å, 2.1 × 100 mm). Water and 0.1% formic acid were used as the A mobile phase, while acetonitrile and 0.1% formic acid were used as the B mobile phase. The gradient system used is 0–1 min (5% B), 1–18 min (5%–45% B), 18–30 min (45%–95% B), 30–33 min (95% B) and 33–35 min (5% B). The column temperature is 30°C. Electrospray ionisation (ESI) with a spray voltage of 3,800 volts (ESI+) and 3,200 volts (ESI–) were used as the ionisation source. The mass analyser was a quadrupole orbitrap (tandem MS/MS), m/z range of 100 Da–1,500 Da. The ionisation energies were set at 18 eV, 35 eV and 53 eV. An injection volume of 2 μL.

Identification of metabolites was carried out using the data in the form of RAW processed with MzMine software. The data were loaded, and feature detection was performed to identify relevant peaks. Parameters for polarity were set to both positive and negative ion modes, with a retention time range of 0 min–35 min and a noise level threshold of 10^4^. The ADAP chromatogram builder was used, with a minimum intensity threshold of 10^6^ and an m/z tolerance of 0.05 m/z or 5 ppm. Chromatogram deconvolution was applied with baseline cut-off algorithm with baseline level 10^6^ and minimum peak height 3 × 10^6^. Followed by gap filling for the m/z and retention time with m/z tolerance of 5 ppm, and grouping of isotopic peaks with retention time tolerance of 0.25 min. All samples were aligned, and metabolites identification was performed using an inhouse metabolite database. Putative identification of metabolites was carried out by comparing the MS and MS-MS spectra against an in-house database and the MetFrag webserver.

### Density Functional Theory (DFT) Studies

Method refers to Rammohan *et al*. (2020), the molecular structure of active antioxidant compounds was optimised and calculated using the DFT B3LYP basis sets 6–31G calculation method with GaussView. The parameters of the molecules reactivity as antioxidants are the highest occupied molecular orbital (HOMO) and lowest unoccupied molecular orbital (LUMO) energyvalues. This energy value is obtained by calculating the system total energy, known as single point energy. Single point energy calculation is carried out on the optimal compound structure. The parameters used are gap energy (ΔE), ionisation energy (I) and electron affinity (A).

### Data and Multivariate Analysis

Analysis of antioxidant capacity data for this study used Minitab software with One Way ANOVA analysis, which was expressed by a *p*-value < 0.05. Next, Tukey’s post hoc test was carried out for tests that were significantly different. Analysis was carried out to find out the treatment that had the most influence on all the tests that had been carried out. All tests were visualised using GraphPad Prism software.

Before carrying out multivariate analysis for UHPLC-Q-Orbitrap HRMS data, pre-processing of the chromatogram with correlation optimized warping (COW) was carried out using The Unscrambler X. Determination of the reference chromatogram is carried out using the similarity index formula and the highest similarity index chromatogram is used as the reference chromatogram. The search for optimal segment length and slack was set with a ratio of 5:1. The ratio starts from 5:1 to 100:20. The highest chromatogram similarity index was selected as data for multivariate analysis. Multivariate PCA, heatmap, and OPLS-DA analyses were performed using the MetaboAnalyst webserver.

To ensure data quality and comparability, a specific preprocessing workflow was implemented: Normalisation by Sum was applied to correct variations between samples, followed by Data Transformation using Log Transformation (log 10) to normalise the data distribution. Finally, Data Scaling was performed using Mean Centering to emphasise the biological variance between the different fractions (*n*-hexane, ethyl acetate, *n*-butanol and water).

Unsupervised PCA was used to visualise the chemical distribution based on fraction polarity. Subsequently, supervised OPLS-DA was utilised to correlate the chemical profiles with antioxidant activity. Models were validated based on R^2^X, R^2^Y and Q^2^ values, where a Q^2^ > 0.5 indicated high predictive reliability. Active antioxidant candidates were selected based on three strict criteria: a Variable Importance in Projection (VIP) score > 1.0, a *p*-value < 0.05, and a Fold Change (FC) > 2.0. Additionally, a hierarchical clustering heatmap was generated using Pearson distance and Ward’s linkage to visualise the relative concentrations of identified metabolites across the fractions.

## RESULTS AND DISCUSSION

### Antioxidant Capacity

Antioxidant capacity was calculated using trolox as the standard curve for all methods. All of the one-way ANOVA tests of *J. gendarussa*’s aerial part fractions using the DPPH, ABTS, FRAP and CUPRAC methods had significantly different results (Fig. 1). The highest antioxidant capacity for the DPPH method was found in the *n*-butanol fraction at 40.5 μmol (trolox equivalent) TE/g dry fraction. However, it was not significantly different from the ethyl acetate fraction. Then, the highest antioxidant capacity of the ABTS and FRAP methods was found in the ethyl acetate fraction at 69.0 and 189 μmol TE/g dry fraction, respectively. Finally, the highest antioxidant capacity of the CUPRAC method was found in the ethyl acetate fraction at 624 μmol TE/g dry fraction. However, it was not significantly different from the *n*-butanol fraction.

It was concluded that active antioxidant compounds are possible in the ethyl acetate or *n*-butanol fraction. These results were compared with research by Zhang *et al*. (2021a), fractionating the aerial parts of *J. gendarussa* using petroleum ether and ethyl acetate, it was found that compounds from the ethyl acetate fraction had the highest antioxidant activity against DPPH with an IC_50_ value of 28.61 μM and the quercetin control was 18.56 μM. The antioxidant values with four different methods, namely DPPH, ABTS, FRAP and CUPRAC, have different results, which can be caused by the uniqueness of each reaction principal method and the content of secondary metabolites contained that have been separated based on different polarities (Sadeer *et al*. 2020).

### Metabolites Identified Using UHPLC-Q-Orbitrap HRMS

Positive and negative ionisation modes of chromatograms were produced from *J. gendarussa*’s aerial parts fractions (Fig. 2a and 2b). There were 37 and 21 metabolites identified in positive and negative ionisation mode, respectively. The distribution of metabolites that have been identified can be seen in the Venn diagram (Fig. 2c and 2d), with detailed metabolite identification results in Tables 1 and 2. The results of metabolite identification using UHPLC-Q-Orbitrap HRMS are putative. Fragmentation is carried out for metabolites with a high-intensity mass spectrum.

A fragmentation example of the major secondary metabolite found in *J. gendarussa* was taken, namely gendarusin A. Gendarusin A belong to flavonoid groups (Ratih *et al*. 2019). The resulting fragmentations of gendarusin A are [C_7_H_5_O_2_]^+^ (m/z 121.0281), [C_17_H_11_O_5_]^+^ (m/z 295.0608), [C_18_H_13_O_6_]^+^ (m/z 325.0720), [C_21_H_17_O_8_]^+^ (m/z 397.0936) and [C_25_H_23_O_11_]^+^ (m/z 499.1240) following previous research (Rahmah 2022). In general, the fragmentation characteristic of flavonoid release H_2_O (Sheng *et al*. 2021). This compound released two H_2_O [M+H-36]^+^ m/z 499.1240, then release C_4_H_7_O_3_ [M+H-102]^+^ m/z 397.0936. Also, Rahmah (2022) reported that fragmentation reaction of gendarusin A released C_8_H_16_O_8_ from [M+H]^+^ 535.14349 → [M+H-C_8_H_16_O_8_]+H^+^ 295.06143, which release arabinose (Table 1).

Compounds identified in positive ionisation mode include phenolics, flavonoids, lignans, coumarins, quinones, terpenoids, fatty acids, steroids, alkaloids and nitrogenous compounds that are following the literature on *J. gendarussa* that have been collected previously (Table 1). There are five phenolics groups identified, including homovanillyl alcohol, protocatechuic acid, cinnamic acid, 3,4-dihydroxy-6-(N-ethylamino) benzamide and sesamol (Calderon *et al*. 2012; Anigboro *et al*. 2020; Rafi *et al*. 2022; Wahyuni *et al*. 2020; Basit *et al*. 2022). Seven flavonoids have been identified, including gendarusin A, gendarusin B, 5-hydroxy-7,8-dimethoxy flavanone, jaceosidin, chrysoeriol, apigenin and schaftoside (Kumar *et al*. 2018; Ratih *et al*. 2019; Rafi *et al*. 2022). Four lignan metabolites were identified, including chinensinaphthol, (+)-lyoniresinol, savinin and justicidone (Perez *et al*. 2004; Bharathi *et al*. 2020; Liu *et al*. 2022). The basic structure of coumarins was identified in *J. gendarussa* (Cassola *et al*. 2019). The quinone group was identified as rapanone (Ratih *et al*. 2019). Three metabolites have been identified in the terpenoid group, including phytol, taraxasterol, and (−)-genpenterpene A (Odokwo & Onifade 2020; Wahyuni *et al*. 2020; Zhang *et al*. 2021b). Seven fatty acids were identified, including 6-(2-Ethyl-5-hydroxy-hexoxy)-6-oxohexanoic acid, (9Z)1,9-heptadecadiene-4,6-diyne-3,8,11-triol, 12-Oxo-phytodienoic acid, gamma-linolenic acid, 9-OxoODE, 13(S)-HODE, and juniperic acid (Calderon *et al*. 2012; Ratih *et al*. 2019). Seven metabolites of alkaloids group have been identified, including flindersine, castanospermine, brazoide C, (8S,9R,9aS, 10aR)-5-Oxo-9-vinyl-1,2,3,8,9,9a,10,10aoctahydro-5H-imidazo[1,2-a] pyrano[4,3-d] pyridin-8-yl-alpha-D-glucopyranoside, justridusamide A, 5H,6H-quinindolin-11-one and 1-Deoxy-1-{methyl[(2-oxo-2,3,4,5-tetrahydro-1H-1-benzazepine-7-yl) carbonyl] amino}hexitol (Kiren *et al*. 2014; Indrayoni *et al*. 2016; Souza *et al*. 2017; Ratih *et al*. 2019; Zhang *et al*. 2021a; Basit *et al*. 2022). Apart from that, a steroid class metabolite was identified, namely stigmasterol (Uddin *et al*. 2011). Finally, nitrogenous compound that was identified was 6-{[(benzyloxy)carbonyl]amino}-6-deoxy-1,2-O-isopropylidene-alpha-D-glucofuranose (Ratih *et al*. 2019).

Compounds identified in negative ionization mode include phenolics, flavonoids, lignans, fatty acids, alkaloids, steroids and nitrogenous compounds (Table 2). The phenolic group identified was sesamol (Basit *et al*. 2022). Six flavonoid metabolites were identified, including gendarusin A, gendarusin B, apigenin, jaceosidin, chryosoeriol and schaftoside (Kumar *et al*. 2018; Ratih *et al*. 2019; Rafi *et al*. 2022). Three lignan metabolites were identified, including taiwanin E methyl ether, medioresinol and (−)-secoisolariciresinol (Xu *et al*. 2019). Four fatty acid metabolites were identified, namely gamma-linolenic acid, 13(S)-HODE, juniperic acid, and 9-oxoODE (Ratih *et al*. 2019). The alkaloids identified were two metabolites, including (8S,9R,9aS, 10aR)-5-oxo-9-vinyl-1,2,3,8,9,9a,10,10aoctahydro-5H-imidazo[1,2-a]pyrano[4,3-d]pyridin-8-yl-alpha-D-glucopyranoside, and 1-deoxy-1-{methyl[(2-oxo-2,3,4,5-tetrahydro-1H-1 - benzazepine-7-yl) carbonyl] amino}hexitol (Souza *et al*. 2017; Ratih *et al*. 2019). In addition, a class of steroids was identified, namely stigmasta-4,22-dien-3-one (Uddin *et al*. 2011). Finally, four nitrogenous metabolites were identified, such as justridusamide A, (3S,5R,6R)-3-Allyl-3-[(S)-hydroxy(4-nitrophenyl) methyl]-5,6-dimethoxy-5,6-dimethyl- 1,4–380,1339dioxan-2-one, methyl 2-[cyclohex-2-en-1-yl(hydroxy) methyl]-3-hydroxy-4-(2-hydroxyethyl)-3-methyl-5-oxoprolinate and 6-{[(Benzyloxy)carbonyl]amino}-6-deoxy-1,2-O-isopropylidene-alpha-D-glucofuranose (Khoo *et al*. 2015; Indrayoni *et al*. 2016; Ratih *et al*. 2019).

### Visualisation of Metabolites Distribution in *J. gendarussa* Fractions

Before PCA was carried out, the chromatogram was first optimised with COW. Visually, the chromatograms can be differentiated by fractionation based on the different levels of solvent polarity (Fig. 2a and 2b). However, PCA will be carried out to reduce the data so that the distribution of metabolites can be seen based on different polarities. The resulting score plot for positive ionisation mode produces data diversity of both PCs values of 79.7% with a PC1 value of 57.8% and PC2 of 21.9% (Fig. 3a). The values of the two PCs show good two-dimensional visualisation because the diversity values of PC1 and PC2 are greater than 70% (Maulana *et al*. 2022). This shows that 79.7% of the data variability can be explained by the peak intensity of *J. gendarussa*’s aerial parts fraction based on different polarities. Likewise, with the score plot from PCA resulting in negative ionisation mode, the diversity of data from both PCs values is 77.2% with a PC1 value of 45.2% and PC2 of 32% (Fig. 3c). Based on the two PCA visualisations (Fig. 3a and 3c), the resulting metabolites from the fractions can be grouped into three large groups, namely polar, semipolar and non-polar metabolites.

Apart from the plot score, there is a Biplot, which combines the plot score and loading plot to see which of the retention time plays a role in sample separation in PCA (Maulana *et al*. 2022). Based on the biplots in Figs. 3b and 3d, information is obtained that the metabolite at a particular retention time in the plot, which points towards the solvent, is the retention time which plays a role in grouping data in PCA for that solvent. Based on the overall PCA visualisation, it can be concluded that fractionation significantly influences the distribution of *J. gendarussa* metabolites.

Furthermore, heatmap analysis will simplify the visualisation of metabolite analysis so that it is easier to understand the separation of each metabolite that has been identified. This analysis can provide a more detailed understanding regarding the grouping of determining metabolites of each sample based on solvent polarity (Yuan *et al*. 2016). In addition, the resulting heatmap proves that metabolites with relatively high concentrations in a solvent are by the isolation of these metabolites in previous research (Figs. 3e and 3f). Gendarusin A and B were isolated in the *n*-butanol fraction (Prajogo *et al*. 2009; 2016). Apigenin was successfully isolated in the ethyl acetate fraction (Kumar *et al*. 2018). Brazoide C was successfully isolated in the ethyl acetate fraction (Souza *et al*. 2017; Zhang *et al*. 2021a). Justridusamide A was isolated in the *n*-butanol fraction (Kiren *et al*. 2014). In addition, in the ethyl acetate fraction, (−)-genpenterpene A was successfully isolated (Zhang *et al*. 2021b).

The observed distribution of these metabolites is closely linked to their chemical structures and the principle of “like dissolves like” across the solvent polarity gradient. The *n*-hexane fraction primarily captured non-polar compounds, including fatty acids like gamma-linolenic acid and 9-OxoODE, as well as steroids such as stigmasterol. In contrast, the ethyl acetate and *n*-butanol fractions were rich in semipolar to polar compounds, specifically flavonoids (for example, gendarusin A, apigenin and jaceosidin) and lignans (for example, savinin). The concentration of these phenolic-rich metabolites in the ethyl acetate and *n*-butanol fractions explains why these two fractions exhibited significantly higher antioxidant capacities in the DPPH, FRAP, and CUPRAC assays compared to the *n*-hexane and water fractions. Furthermore, the heatmap analysis (Figs. 3e and 3f) reveals that although some major metabolites, such as gendarusin A, were present in both semipolar fractions, their relative abundances differed, suggesting that the synergistic effect of the specific metabolite matrix in each fraction plays a vital role in their overall biological potential.

### Prediction of Potential Compounds as Antioxidant

OPLS-DA is a chemometric method used to explain variations between sample groups and eliminate data irrelevant to the information needed through orthogonalisation. OPLS-DA can more easily exclude independent variables unrelated to classification and filter characteristic variables in the sample. OPLS-DA can also interpret models more quickly because it focuses on predictions, namely discriminants, that can be used in discriminant method to get the best classification results (Arendse *et al*. 2018).

The OPLS-DA model was evaluated using parameters consisting of cumulative values of R^2^X, R^2^Y and Q^2^. R^2^X explains the variation of variable X, and R^2^Y explains the variation of variable Y, which consists of predictive and orthogonal components. Q^2^ shows the variation of Y predicted by model X in that component. The OPLS-DA model is said to have good predictive ability if it produces a Q^2^ value > 0.5 (Zhang *et al*. 2019). These identified metabolites will be correlated with antioxidant capacity to predict the *J. gendarussa*’s active antioxidant compounds with OPLS-DA. All OPLS-DA models for DPPH, ABTS, FRAP and CUPRAC produce Q^2^ values > 0.5, meaning the resulting prediction models are good. Example the resulting OPLS-DA models of DPPH are shown in Figs. 4a and 4b.

OPLS-DA was created using peak area variables from all m/z detected for identified and unknown metabolites and antioxidant capacity values. The antioxidant capacity value is classified by creating an interval between the lowest and the highest antioxidant capacity value (Tunnisa *et al*. 2022). The OPLS-DA score plot showed that the two groups were separated based on their antioxidant capacity. Example on DPPH method, the left quadrant shows the active group with high antioxidant capacity values consisting of the ethyl acetate and *n*-butanol fractions. In contrast, the right quadrant consists of the inactive *n*-hexane and water fractions with low antioxidant capacity values (Figure 4a). The score plot also shows that the model succeeded in grouping samples based on their antioxidant capacity.

Compounds that have the potential as antioxidants are predicted using S-plot. S-plot, the X-axis represents the variables contribution to the variance of the observed samples, while the Y-axis represents the correlation between samples and the results reliability (Xu *et al*. 2021). A combination of S-plot and several variables determine the compounds with the most probability act as an antioxidant. Variables were selected based on several criteria, namely p-value < 0.05, fold change > 2 and VIP > 1. VIP reflects the loading weight for each molecular weight variable and the response variability described by this value. Fold change is a measure that describes the change in quantity between the original measurement and the subsequent measurement (Luo *et al*. 2023). Based on these criteria, the predicted antioxidant active compounds will be in the lower left or upper right quadrant, fulfilling all the criteria. The plot score shows that the left quadrant is the group with high antioxidant capacity values, so that in the S-plot the lower left quadrant is the predicted area for active antioxidant compounds (Figure 4b). A summary of the active compound prediction results is attached in Table 3.

Based on this table, gendarusin A and five unknown compounds were found as the predicted active antioxidant compounds using the DPPH, ABTS and CUPRAC methods. Then, using the FRAP method, 3,4-dihydroxy-6-(N-ethylamino) benzamide, 6-{[(Benzyloxy)carbonyl]amino}-6-deoxy-1,2-O-isopropylidene-alpha-D- glucofuranose, methyl 2-[cyclohex-2-en-1-yl(hydroxy) methyl]-3-hydroxy-4-(2-hydroxyethyl)-3-methyl-5-oxoprolinate, sesamol, and justridusamide A as well as nine unknown compounds as the predicted active antioxidant compounds. There were six identified metabolites and 14 unknown predicted as the active antioxidant compounds.

Each antioxidant method has its unique reaction. The FRAP method produces different results, which several factors can cause. FRAP obtains different results due to non-specific reactions to non-antioxidant compounds (Sadowska-Bartosz & Bartosz 2022). Then, the FRAP method reacts to a pH of 3.6, which is less representative of the biological situation, while the DPPH, ABTS, and CUPRAC methods have a reaction pH of 7.0 (Sadeer *et al*. 2020). Apart from that, the FRAP method also reacts weakly with flavonoid compounds. Therefore, gendarusin A is not predicted to be active in the FRAP method (Sadeer *et al*. 2020).

Unknown compounds identified as active antioxidants have the potential to be isolated and might be new chemical structure compounds to be obtained. Therefore, heatmap analysis was carried out specifically for the unknown compounds shown in Figure 4c. This was done to identify potential fractions for isolation in further research. Based on the heatmap (Figure 4c), the ethyl acetate fraction is dominant for isolation due to the high relative concentration of unknown compounds predicted to be active antioxidants. The six metabolites that have been identified in *J. gendarussa* will be subjected to a DFT study to prove and strengthen the predicted results are active antioxidant compounds.

### Density Functional Theory (DFT) of *J. gendarussa* Active Antioxidant Compounds

Computational chemistry methods for drug development are commonly used as a complement to *in vitro* and *in vivo* by medicinal chemistry researchers in exploring and finding lead compounds and modifying them into drug candidates (Salman *et al*. 2021). One approach that can be used to evaluate the ability of a compound as an antioxidant is density functional theory (DFT) studies. DFT is a highly accurate computational chemistry method that can provide detailed information about molecular properties, including reactivity and thermodynamics. One of the main properties obtained from DFT calculations is the system total energy, which is often called single point energy. Single point energy calculations provide insight into the stability, reactivity, and various properties of molecules. The highest occupied molecular orbital (HOMO) and lowest unoccupied molecular orbital (LUMO) energy values are the parameters used in this research (Kaddouri *et al*. 2020).

The HOMO energy represents the highest energy level occupied by an electron in a molecule. A lower HOMO energy indicates a greater electron availability, making it more reactive in nucleophilic reactions. LUMO energy represents the lowest unoccupied energy level in a molecule, indicating its ability to accept electrons. A high LUMO energy indicates a good electron acceptor and tends to be more reactive in electrophilic reactions. Based on HOMO and LUMO energy, the ionisation energy and electron affinity of a molecule can be calculated. Ionisation energy is the amount of energy required to release one outermost electron from an atom. The smaller the ionisation energy value, the easier it is for electrons to escape or move out of the path, so the molecule is said to be more reactive. Electron affinity is the energy change that occurs when an atom or molecule gains electrons. Higher electron affinity tends to make molecules more reactive (Azuxetullatif *et al*. 2020; Rammohan *et al*. 2020). The conclusion is that the best compound as an antioxidant is taken from the smallest energy gap (Islam 2015).

The energy gap is defined as the difference between the HOMO and LUMO energy values. The smaller the energy gap, the easier it is for electrons to be excited and the easier it is for a molecule to react. This is related to the antioxidant mechanism of the DPPH, ABTS, FRAP and CUPRAC methods. A small energy gap characterises compounds that are reactive to radicals, indicating good antioxidant activity (Sidiq 2021).

Table 4 summarises all DFT results. Based on this table, the smallest ionisation energy, largest electron affinity and smallest gap energy parameters are found in the compound 6-{[(Benzyloxy) carbonyl]amino}-6-deoxy-1,2-O-isopropylidene-alpha-D-glucofuranose. The order of compounds based on the highest antioxidant power are 6-{[(Benzyloxy) carbonyl]amino}-6-deoxy-1,2-O-isopropylidene-alpha-D-glucofuranose > gendarusin A > trolox > 3,4-Dihydroxy-6- (N-ethylamino) benzamide > justridusamide A > sesamol > Methyl 2-[cyclohex-2-en-1-yl(hydroxy) methyl]-3-hydroxy-4-(2-hydroxyethyl)-3-methyl-5-oxoprolinate. Research by Srisayam *et al*. (2014), proves that sesamol has and IC_50_ value of 14.48 μM throught DPPH method. This proves that the prediction of active antioxidant compounds using OPLS-DA succeeded in obtaining two compounds with an energy gap smaller than the standard used to determine antioxidant capacity *in vitro*. Furthermore, isolation can be carried out to prove its antioxidant ability using metabolomics and density functional theory approaches.

## CONCLUSION

The ethyl acetate and *n*-butanol fractions are the fractions that have the highest antioxidant capacity in the DPPH, ABTS, FRAP and CUPRAC methods. There were 37 and 21 metabolites identified in positive and negative ionisation using UHPLC-Q-Orbitrap HRMS, respectively. PCA and heatmap succeeded in differentiating metabolites based on polarity. OPLS-DA succeeded in identifying six active metabolites and 14 unknown compounds that had the potential to be isolated in the ethyl acetate fraction. The DFT study proves that the best antioxidant compound is 6-{[(benzyloxy) carbonyl]amino}-6-deoxy-1,2-O-isopropylidene-alpha-D-glucofuranose and gendarusin A compared trolox as a standard. Therefore, those two compounds are the best antioxidant compounds.

## Figures and Tables

**FIGURE 1:**
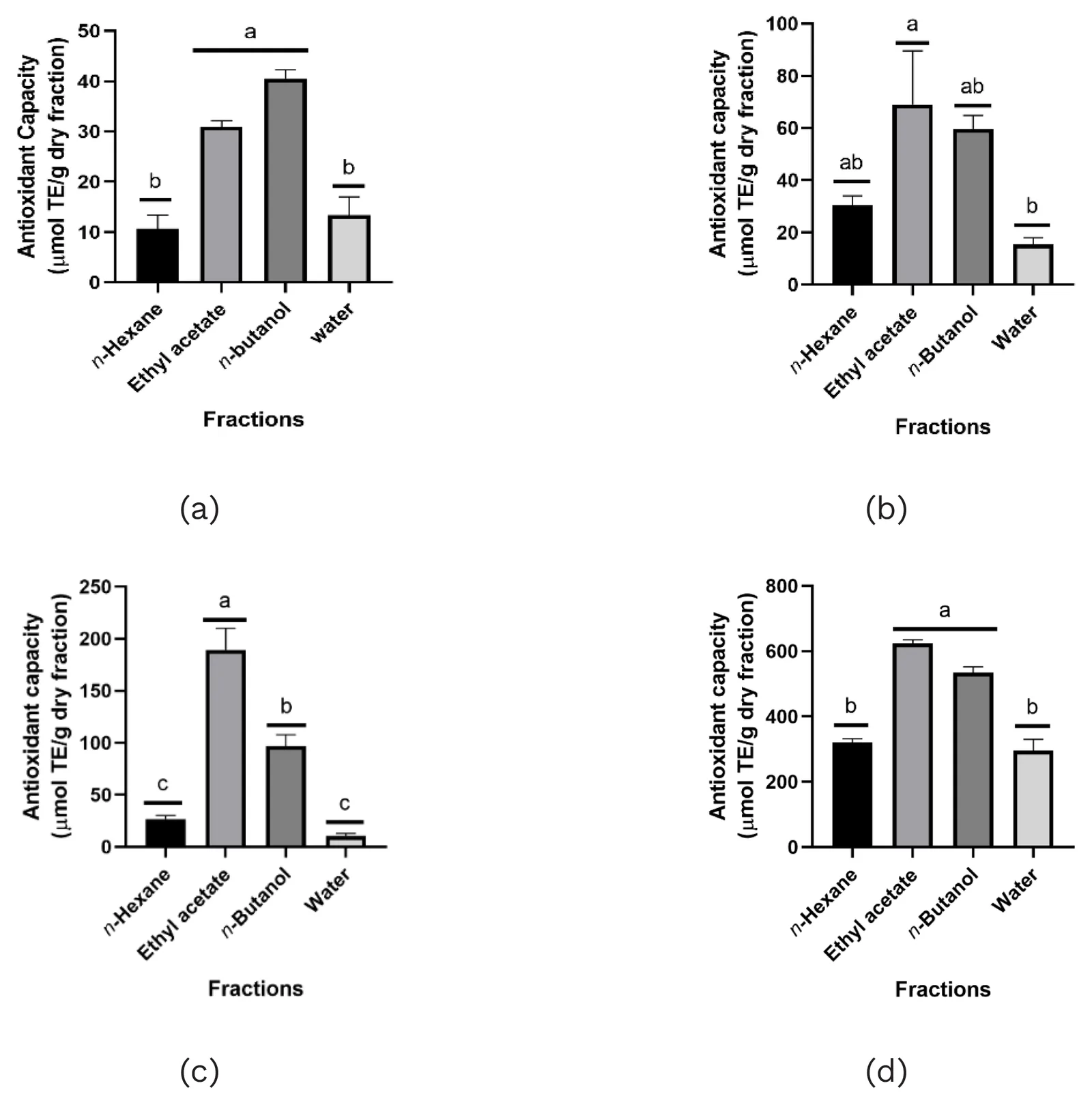
Antioxidant capacity of *J. gendarussa*’s aerial parts fraction: (a) DPPH (b) ABTS (c) FRAP and (d) CUPRAC method. Each value is presented as the mean ± standard error of mean (SEM). The mean value in each column marked with a different letter is significantly different at *p* < 0.05 or the 95% confidence level.

**FIGURE 2:**
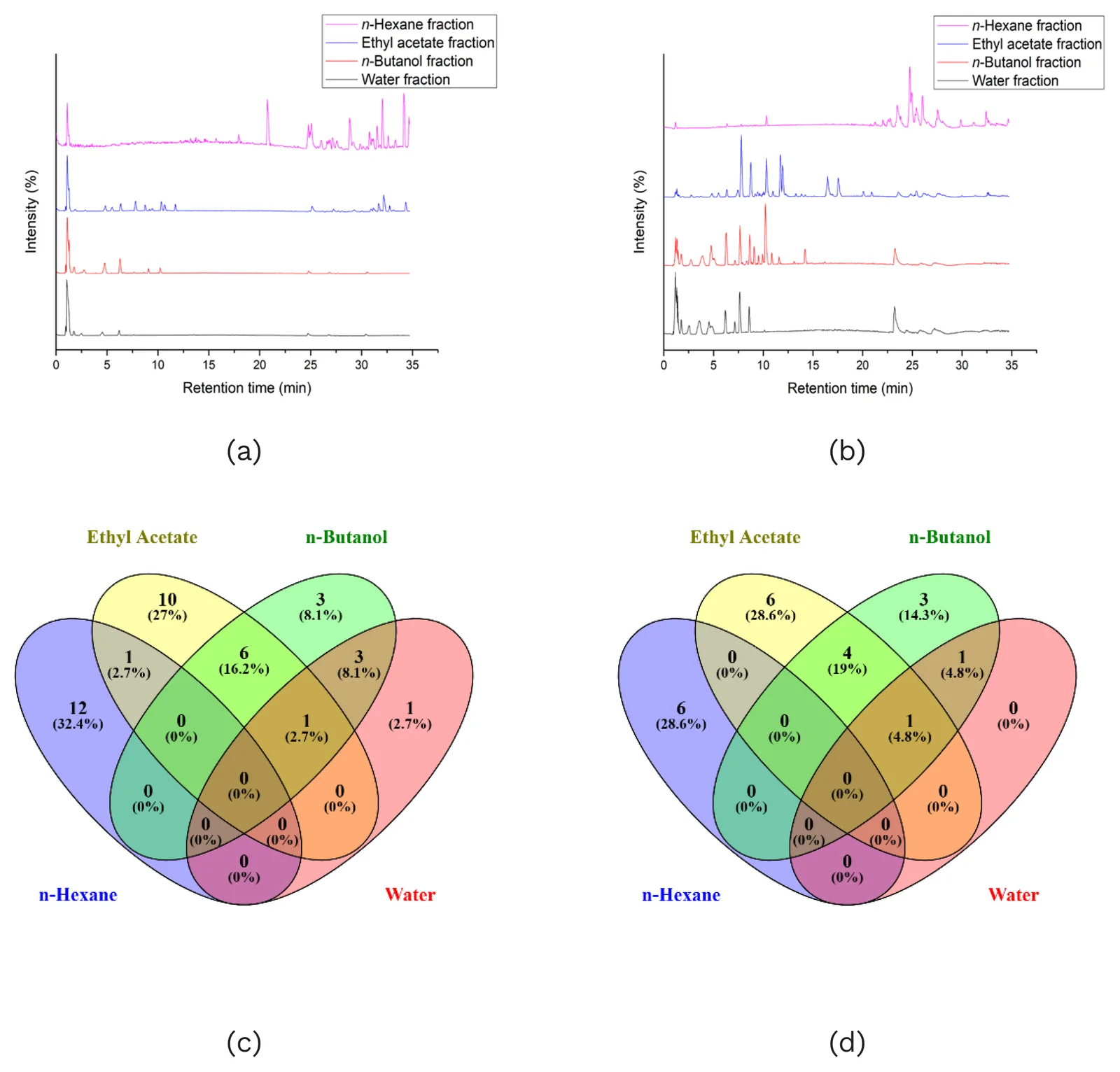
Fraction chromatogram. (a) Positive ionisation mode; (b) negative ionisation mode. Venn diagram of identified compounds: (c) Positive ionisation mode; (d) negative ionisation mode.

**FIGURE 3:**
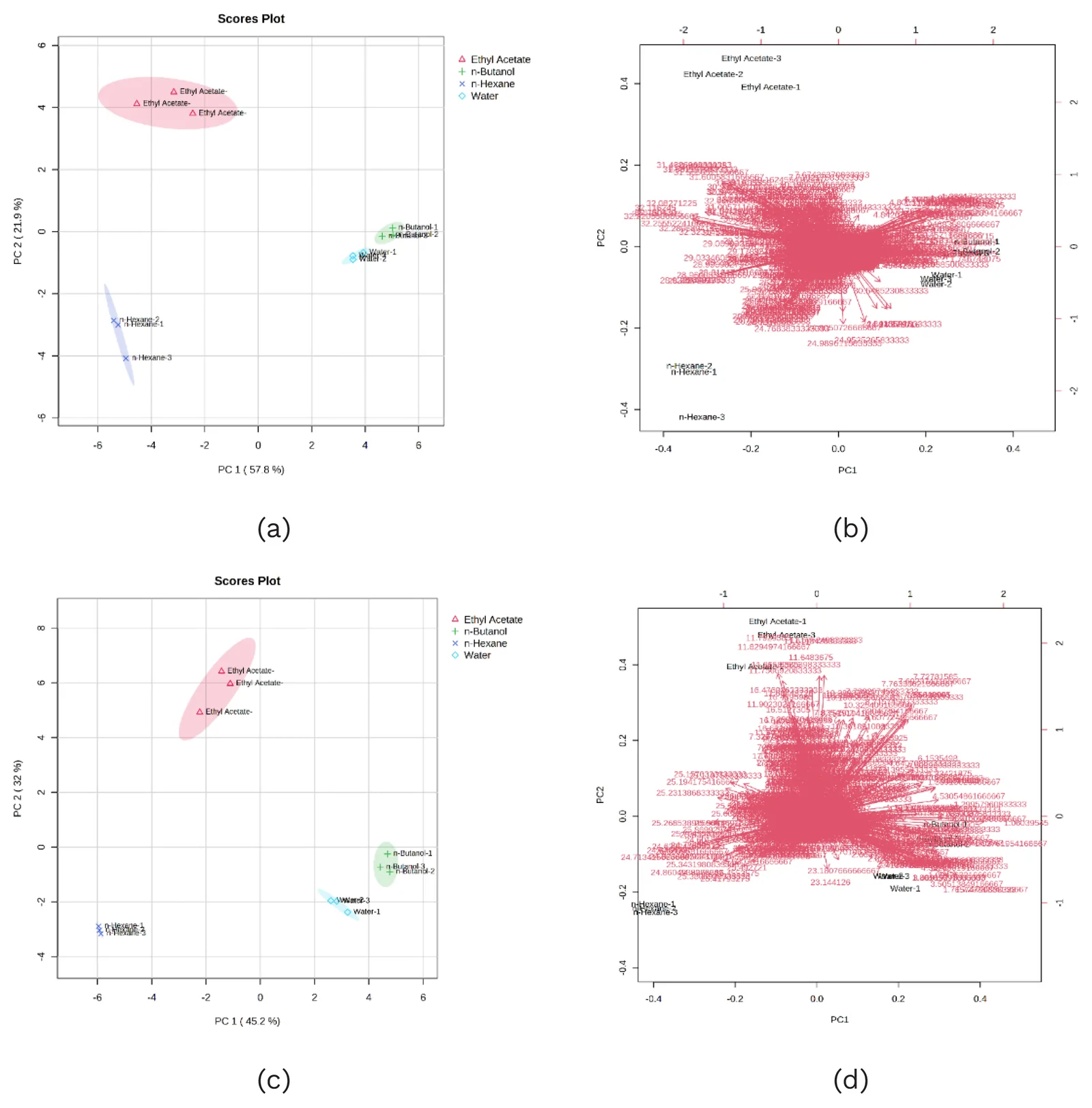

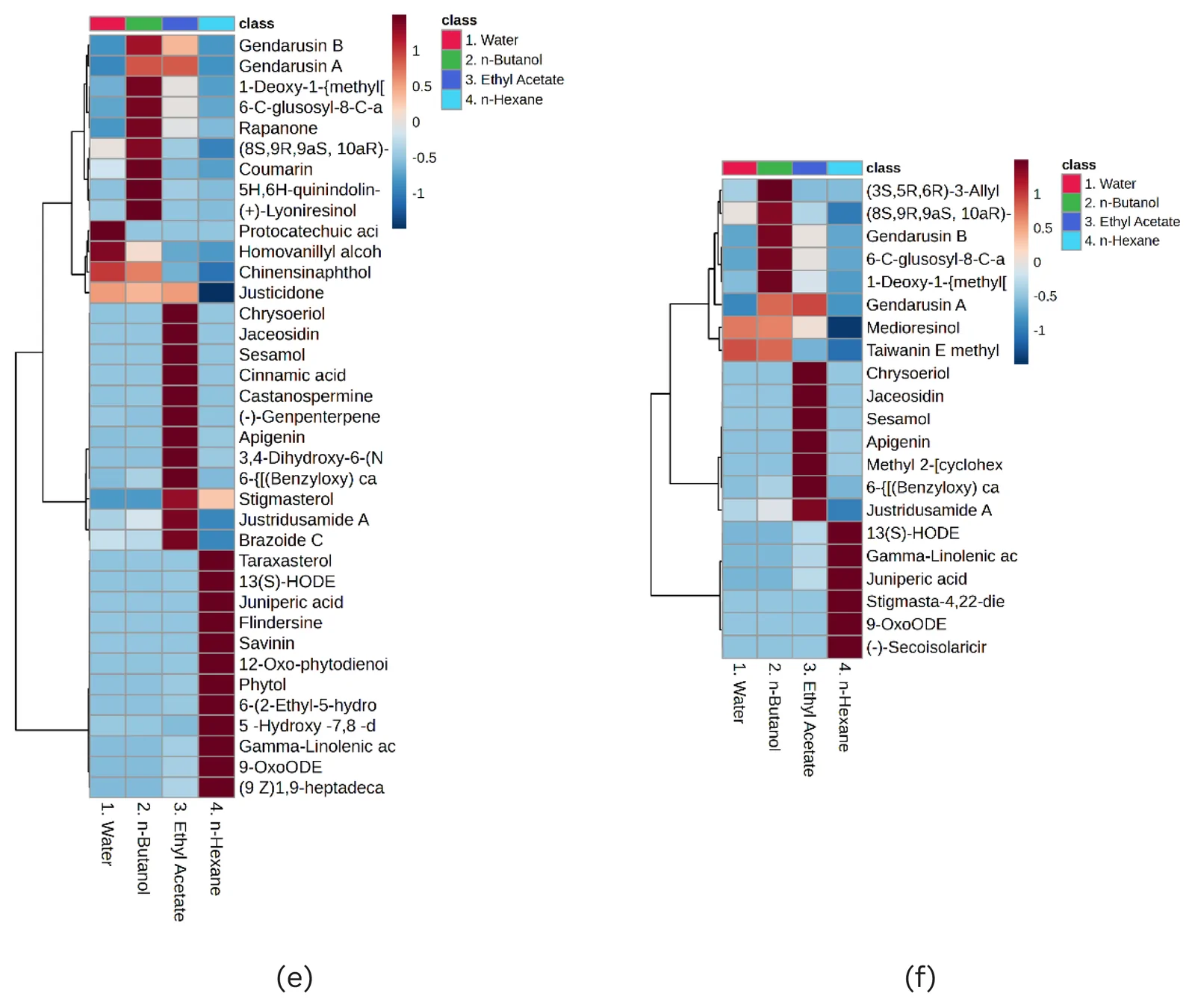
PCA of *J. gendarussa* fraction. (a) Positive ionisation plot score; (b) positive ionisation biplot; (c) negative ionisation plot score; and (d) negative ionisation biplot. Heatmap of metabolites identified in *J. gendarussa*’s aerial parts fraction. (e) Positive ionization; and (f) negative ionisation.

**FIGURE 4:**
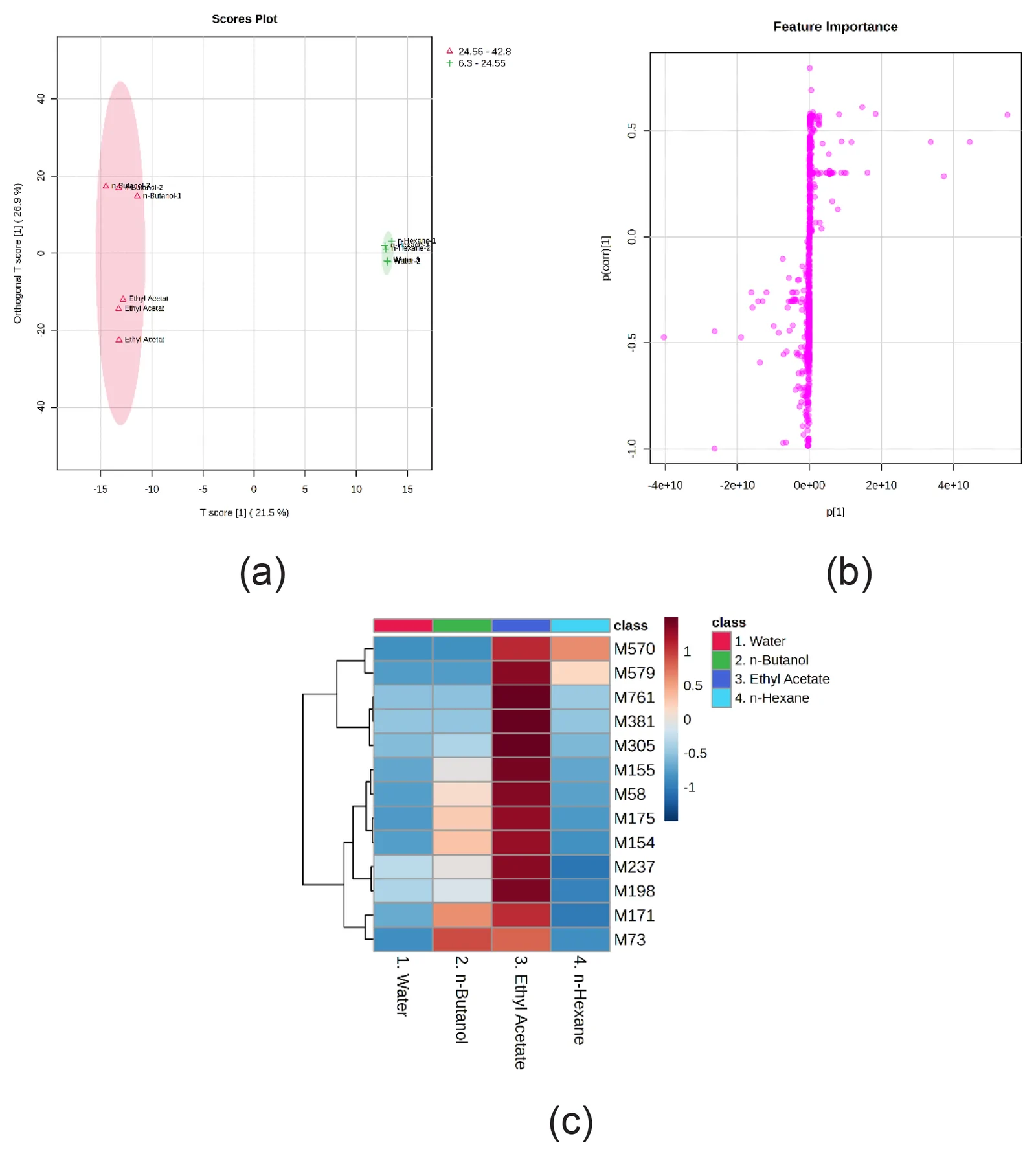
OPLS-DA of (a) DPPH method score plot; (b) DPPH method S-plot; and (c) Heatmap of unknown compounds predicted as active antioxidant.

**TABLE 1:** Identified metabolites with positive ionisation in *J. gendarussa*’s aerial parts fraction.

No.	Rt (minute)	Name	Molecular formula	MS and MS-MS	Group	A	B	C	D
1	0.9468	Homovanillyl alcohol	C_9_H_12_O_3_	[M-H]^+^ 169.0363	Phenolics			√	√
2	1.1019	Chinensinaphthol	C_21_H_16_O_7_	[M-H]^+^ 381.0783	Lignans			√	√
3	1.1973	Justicidone	C_20_H_12_O_7_	[M-H]^+^ 365.1045	Lignans		√	√	√
4	1.1425	Coumarin	C_9_H_6_O_2_	[M-H]^+^ 147.0378 [C_5_H_5_]^+^ 65.0393 [C_7_H_7_]^+^ 91.0542 [C_8_H_7_O]^+^ 119.049	Coumarins			√	
5	1.5067	Cinnamic acid	C_9_H_8_O_2_	[M-H]^+^ 149.0293	Phenolics		√		
6	1.7891	Brazoide C	C_18_H_26_N_2_O_9_	[M-H]^+^ 415.1692	Alkaloids		√		
7	4.7389	(+)-Lyoniresinol	C_22_H_28_O_8_	[M-H]^+^ 421.1466	Lignans			√	
8	6.2850	(8S,9R,9aS,10aR)-5-Oxo-9-vinyl-1,2,3,8,9,9a,10,10aoctahydro-5H-imidazo[1,2-a] pyrano [4,3-d] pyridin-8-yl-alpha-D-glucopyranoside	C_18_H_26_N_2_O_8_	[M-H]^+^ 399.1745	Alkaloids			√	√
9	6.6963	Rapanone	C_19_H_30_O_4_	[M-H]^+^ 323.1622 [C_10_H_9_O_3_]^+^ 177.0537	Cuinons		√	√	
10	7.7125	Justridusamide A	C_17_H_23_NO_8_	[M-H]^+^ 370.1469 [C_7_H_8_N]^+^ 106.0651 [C_7_H_12_O_4_]^+^ 160.0748 [C_11_H_10_NO_2_]^+^188.0706 [C_11_H_12_NO_3_]^+^ 206.0812	Nitrogenous compound		√		
11	8.7758	(−)-Genpenterpene A	C_12_H_16_O_3_	[M-H]^+^ 209.0983	Terpenoids		√		
12	9.1025	1-Deoxy-1-{methyl[(2-oxo-2,3,4,5-tetrahydro-1H-1-benzazepin-7-yl) carbonyl] amino}hexitol	C_18_H_26_N_2_O_7_	[M-H]^+^ 383.1876 [C_4_H_6_NO]^+^ 84.0445 [C_7_H_8_N]^+^ 106.0951 [C_10_H_10_N]^+^ 144.0810 [C_12_H_13_N_2_O_2_]^+^ 217.0979 [C_12_H_15_N_2_O_3_]^+^ 235.1079	Alkaloids		√	√	
13	9.1026	5H,6H-quinindolin-11-one	C_15_H_10_N_2_O	[M-H]^+^ 235.0940 [C_7_H_8_N]^+^ 106.0650 [C_8_H_8_N]^+^ 118.0655 [C_9_H_8_N]^+^ 130.0648 [C_11_H_11_N_2_]^+^ 171.0922	Alkaloids			√	
14	9.3644	Castanospermine	C_8_H_15_NO_4_	[M-H]^+^ 190.0753	Alkaloids		√		
15	9.4008	6-C-glusosyl-8-C-arabinosyl apigenin or Schaftoside	C_26_H_28_O_14_	[M-H]^+^ 565.1540	Flavonoids		√	√	
16	10.2375	Gendarusin A	C_25_H_26_O_13_	[M-H]^+^ 535.1431 [C_7_H_5_O_2_]^+^ 121.0281 [C_17_H_11_O_5_]^+^ 295.0608 [C_18_H_13_O_6_]^+^ 325.0720 [C_21_H_17_O_8_]^+^ 397.0936 [C_25_H_23_O_11_]^+^ 499.1240	Flavonoids		√	√	
17	10.5596	3,4-Dihydroxy-6-(N-ethylamino) benzamide	C_9_H_12_N_2_O_3_	[M-H]^+^ 197.1004	Phenolics		√		
18	10.8853	Gendarusin B	C_25_H_26_O_13_	[M-H]^+^ 535.1403 [C_7_H_5_O_2_]^+^ 121.0283 [C_14_H_15_O_7_]^+^ 295.0820 [C_18_H_13_O_6_]^+^ 325.0724 [C_25_H_23_O_11_]^+^ 499.1245	Flavonoids		√	√	
19	11.6129	6-{[(Benzyloxy) carbonyl]amino}-6-deoxy-1,2-O-isopropylidene-alpha-D-glucofuranose	C_17_H_23_NO_7_	[M-H]^+^ 354.1511	Nitrogenous compound		√		
20	12.7150	Protocatechuic acid	C_7_H_6_O_4_	[M-H]^+^ 154.9929	Phenolics				√
21	14.0909	Flindersine	C_14_H_13_NO_2_	[M-H]^+^ 228.1047	Alkaloids	√			
22	16.3481	Apigenin	C_15_H_10_O_5_	[M-H]^+^ 271.0777 [C_5_H_7_]^+^ 67.0549 [C_8_H_7_O]^+^ 119.0494 [C_7_H_5_O_4_]^+^ 153.0183	Flavonoids		√		
23	16.7338	Jaceosidin	C_17_H_14_O_7_	[M-H]^+^ 330.2986	Flavonoids		√		
24	16.7975	Chrysoeriol	C_16_H_12_O_6_	[M-H]^+^ 301.0743	Flavonoids		√		
25	20.6770	Savinin	C_20_H_16_O_6_	[M-H]^+^ 353.1135	Lignans	√			
26	20.8683	Sesamol	C_7_H_6_O_3_	[M-H]^+^ 139.0103	Phenolics		√		
27	21.5430	6-(2-Ethyl-5-hydroxy-hexoxy)-6-oxohexanoic acid	C_14_H_26_O_5_	[M-H]^+^ 275.1927	Fatty acids	√			
28	24.6151	(9Z)1,9-heptadecadiene-4, 6-diyne-3,8,11-triol	C_17_H_24_O_3_	[M-H]^+^ 277.2155 [C_5_H_7_]^+^ 67.0545 [C_6_H_7_]^+^ 79.0542	Fatty acids	√			
29	25.4601	12-Oxo-phytodienoic acid	C_18_H_28_O_3_	[M-H]^+^ 293.2000 [C_4_H_7_]^+^ 55.0546 [C_5_H_7_]^+^ 67.0545 [C_12_H_15_]^+^ 159.1160 [C_18_H_25_O]^+^ 257.1914 [C_18_H_27_O_2_]^+^ 275.1995	Fatty acids	√			
30	26.0763	Gamma-Linolenic acid	C_18_H_30_O_2_	[M-H]^+^ 279.2274 [C_4_H_7_]^+^ 55.0546 [C_5_H_7_]^+^ 67.0544 [C_6_H_9_]^+^ 81.0705 [C_7_H_11_]^+^ 95.0854 [C_8_H_13_]^+^ 109.1006 [C_9_H_15_]^+^ 123.1167 [C_13_H_21_O_2_]^+^ 209.1523 [C_14_H_23_O_2_]^+^ 223.1680 [C_18_H_29_O]^+^ 261.2198	Fatty acids	√			
31	26.6814	9-OxoODE	C_18_H_30_O_3_	[M-H]^+^ 295.2051 [C_4_H_7_]^+^ 55.0546 [C_5_H_7_]^+^ 67.0545 [C_5_H_5_O]^+^ 81.0699 [C_10_H_15_O]^+^ 151.1111 [C_12_H_17_]^+^ 161.1322 [C_13_H_19_O_2_]^+^ 207.1364	Fatty acids	√			
32	27.4184	5-Hydroxy-7,8-dimethoxy flavanone	C_17_H_16_O_5_	[M-H]^+^ 301.1457	Flavonoids	√			
33	27.7739	13(S)-HODE	C_18_H_32_O_3_	[M-H]^+^ 297.2426 [C_6_H_9_]^+^ 81.07 [C_7_H_11_]^+^ 95.0856 [C_8_H_13_]^+^ 109.1009 [C_8_H_13_O]^+^ 125.0962 [C_10_H_15_]^+^ 135.1173 [C_18_H_29_O]^+^ 261.2204 [C_18_H_31_O_2_]^+^ 279.2310	Fatty acids	√			
34	31.5955	Juniperic acid	C_16_H_32_O_3_	[M-H]^+^ 273.2534	Fatty acids	√			
35	31.6745	Stigmasterol	C_29_H_48_O	[M-H]^+^ 413.3213 [C_7_H_11_]^+^ 95.0859 [C_10_H_13_]^+^ 133.1000 [C_12_H_15_]^+^ 159.1169 [C_15_H_19_]^+^ 199.1486	Steroids	√			
36	32.3087	Taraxasterol	C_30_H_50_O	[M-H]^+^ 427.3765	Terpenoids	√			
37	32.7056	Phytol	C_20_H_40_O	[M-H]^+^ 297.3145 [C_4_H_9_]^+^ 57.0703 [C_5_H_9_]^+^ 69.0704 [C_6_H_11_]^+^ 83.0860 [C_7_H_13_]^+^ 97.1017 [C_9_H_17_]^+^ 125.1322 [C_10_H_19_]^+^ 139.1497	Terpenoids	√			

*Notes*: A = *n*-hexane fraction; B = ethyl acetate fraction; C = *n*-butanol fraction; D = water fraction

**TABLE 2:** Identified metabolites with negative ionisation in *J. gendarussa*’s aerial parts fraction.

No	Rt (minute)	Name	Molecular formula	MS and MS-MS	Group	A	B	C	D
1	1.1531	Taiwanin E methyl ether	C_21_H_14_O_7_	[M-H]^−^ 377.0855	Lignans			√	√
2	1.1558	Medioresinol	C_21_H_24_O_7_	[M-H]^−^ 387.1146	Lignans		√	√	√
3	6.2852	(8S,9R,9aS, 10aR)-5-Oxo-9-vinyl-1,2,3,8,9,9a,10,10aoctahydro-5H-imidazo[1,2-a] pyrano [4,3-d] pyridin-8- alpha-D-glucopyranoside	C_18_H_26_N_2_O_8_	[M-H]^−^ 397.1619	Alkaloids			√	
4	7.7093	Justridusamide A	C_17_H_23_NO_8_	[M-H]^−^ 368.1358 [C_4_H_5_O_3_]^−^101.0230 [C_4_H_7_O_4_]^−^ 119.0345 [C_6_H_11_O_5_]^−^ 163.0603 [C_11_H_10_NO_3_]^−^ 204.067 [C_11_H_12_NO_4_]^−^ 222.0756	Nitrogenous compound		√		
5	8.3808	(3S,5R,6R)-3-Allyl-3-[(S)-hydroxy(4-nitrophenyl) methyl]-5,6-dimethoxy-5,6-dimethyl-1,4–380,1339dioxan-2-one	C_18_H_23_NO_8_	[M-H]^−^ 380.1381	Nitrogenous compound			√	
6	9.1028	1-Deoxy-1-{methyl[(2-oxo-2,3,4,5-tetrahydro-1H-1- benzazepin-7-yl) carbonyl] amino}hexitol	C_18_H_26_N_2_O_7_	[M-H]^−^ 381.1668	Alkaloids			√	
7	9.1482	6-C-glusosyl-8-C-arabinosyl apigenin or Schaftoside	C_26_H_28_O_14_	[M-H]^−^ 563.1406	Flavonoids		√	√	
8	10.2012	Gendarusin A	C_25_H_26_O_13_	[M-H]^−^ 533.1308	Flavonoids		√	√	
9	10.8793	Gendarusin B	C_25_H_26_O_13_	[M-H]^−^ 533.1306	Flavonoids		√	√	
10	11.5937	Sesamol	C_7_H_6_O_3_	[M-H]^−^ 137.0402 [C_6_H_5_O]^−^ 93.0336	Phenolics		√		
11	11.6158	6-{[(Benzyloxy) carbonyl]amino} -6-deoxy-1,2-O-isopropylidene-alpha-D-glucofuranose	C_17_H_23_NO_7_	[M-H]^−^ 352.1414	Nitrogenous compound		√		
12	16.3587	Methyl 2-[cyclohex-2-en-1-yl(hydroxy) methyl]-3-hydroxy-4-(2- hydroxyethyl)-3-methyl-5-oxoprolinate	C_16_H_25_NO_6_	[M-H]^−^ 327.2150	Nitrogenous compound		√		
13	16.4997	Apigenin	C_15_H_10_O_5_	[M-H]^−^ 269.0524	Flavonoids		√		
14	16.7387	Jaceosidin	C_17_H_14_O_7_	[M-H]^−^ 329.0441	Flavonoids		√		
15	16.8444	Chrysoeriol	C_16_H_12_O_6_	[M-H]^−^ 299.0760	Flavonoids		√		
16	22.0038	Gamma-Linolenic acid	C_18_H_30_O_2_	[M+H]^−^ 277.1847	Fatty acids	√			
17	24.6149	(−)-Secoisolariciresinol	C_20_H_26_O_6_	[M-H]^−^ 361.2199 [C_6_H_7_O]^−^ 95.0488 [C_7_H_9_O]^−^ 109.0655 [C_9_H_15_O_3_]^−^ 171.1023 [C_12_H_19_O_3_]^−^ 211.1335 [C_15_H_18_O_3_]^−^ 246.1255	Lignans	√			
18	25.9558	13(S)-HODE	C_18_H_32_O_3_	[M-H]^−^ 295.2273 [C_7_H_13_O]^−^ 113.0963 [C_12_H_19_O_2_]^−^ 195.1383	Fatty acids	√			
19	26.2882	9-OxoODE	C_18_H_30_O_3_	[M-H]^−^ 293.1852 [C_2_H_3_O_2_]^−^ 59.0129 [C_7_H_13_O]^−^ 113.0964 [C_10_H_15_O_2_]^−^ 167.1065 [C_10_H_17_O_3_]^−^ 185.1175 [C_17_H_29_O]^−^ 249.2233	Fatty acids	√			
20	29.7393	Juniperic acid	C_16_H_32_O_3_	[M-H]^−^ 271.2267 [C_15_H_29_O]^−^ 225.2206	Fatty acids	√			
21	32.3554	Stigmasta-4,22-dien-3-one	C_29_H_46_O	[M-H]^−^ 409.3041	Steroids	√			

*Note*: A = *n*-hexane fraction; B = ethyl acetate fraction; C = *n*-butanol fraction; D = water fraction

**TABLE 3:** Summary of predicted active antioxidant compounds with OPLS-DA.

Antioxidant method	Metabolites code	ESI	Metabolite name	Molecular weight	VIP value	Fold change
DPPH, ABTS, CUPRAC	M73	+	Unknown	123.0431	2.15	1201.40
	M745	−	Gendarusin A	533.1308	2.08	19.27
	M277	+	Gendarusin A	535.1431	2.09	18.66
	M171	+	Unknown	376.1307	2.01	9.97
	M154	+	Unknown	376.1258	1.92	32.21
	M58	+	Unknown	136.0552	1.80	82.31
	M175	+	Unknown	336.1271	1.77	196.84
FRAP	M381	+	Unknown	110.0242	1.26	435.24
	M440	+	Sesamol	139.0103	1.34	382.53
	M579	+	Unknown	609.2669	1.36	6.66
	M289	+	3,4-Dihydroxy-6-(N-ethylamino) benzamide	197.1004	1.69	6.28
	M783	−	Methyl 2-[cyclohex-2-en-1-yl(hydroxy) methyl]-3-hydroxy-4-(2-hydroxyethyl)-3-methyl-5-oxoprolinate	327.2150	1.64	90.12
FRAP	M715	−	Justridusamide A	368.1351	1.54	4.30
	M756	−	6-{[(Benzyloxy) carbonyl]amino} -6-deoxy-1,2-O-isopropylidene-alpha-D-glucofuranose	352.1414	1.41	9.38
	M202	+	Justridusamide A	370.1469	1.55	5.15
	M761	−	Unknown	187.1061	1.69	71.82
	M305	+	Unknown	206.0759	1.65	23.08
	M198	+	Unknown	206.0786	1.54	4.65
	M302	+	6-{[(Benzyloxy) carbonyl]amino} -6-deoxy-1,2-O-isopropylidene-alpha-D-glucofuranose	354.1511	1.66	29.32
	M753	−	Sesamol	137.0402	1.68	249.66
	M155	+	Unknown	336.1272	1.53	9.42
	M570	+	Unknown	609.2676	1.18	3.96
	M154	+	Unknown	376.1258	1.44	5.06
	M237	+	Unknown	206.0769	1.46	3.91

**TABLE 4:** DFT active antioxidant compounds and Trolox.

Compound name	I (eV)	A (eV)	Molecular orbital
6-{[(Benzyloxy) carbonyl]amino}-6-deoxy-1,2-O-isopropylidene-alpha-D-glucofuranose	5.8635	3.4885	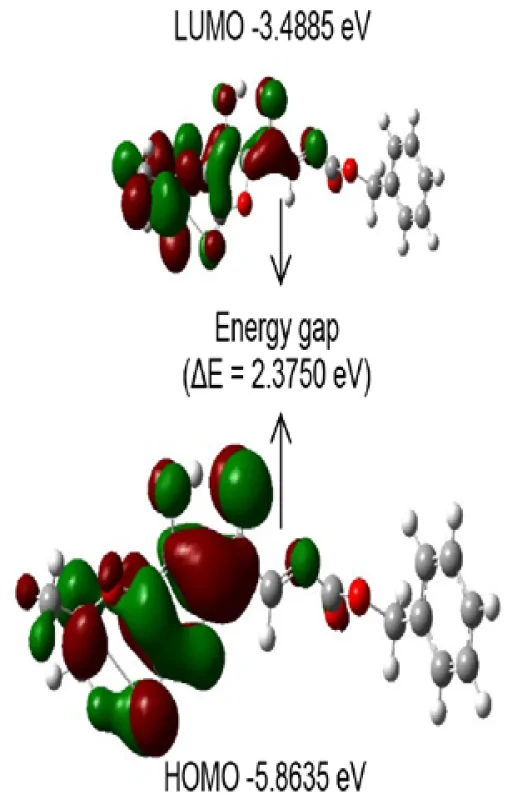
3,4-Dihydroxy-6-(N-ethylamino) benzamide	5.9552	1.101	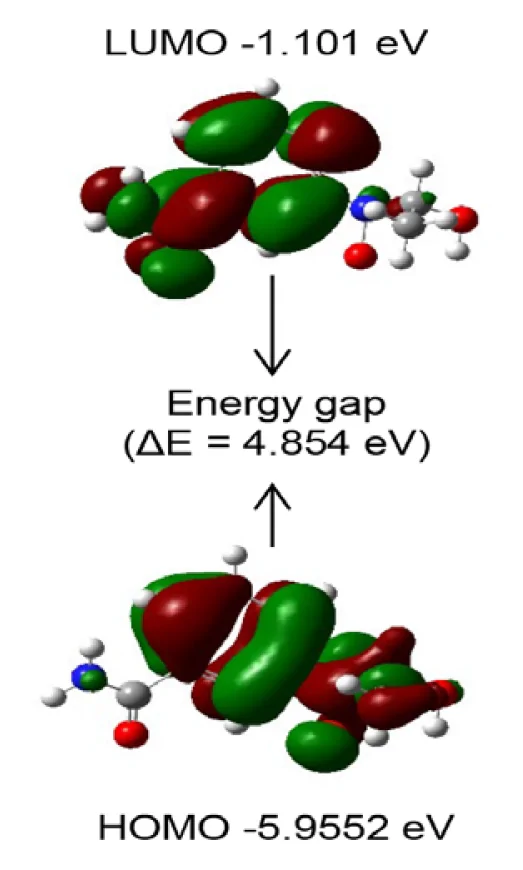
Gendarusin A	6.2409	2.160	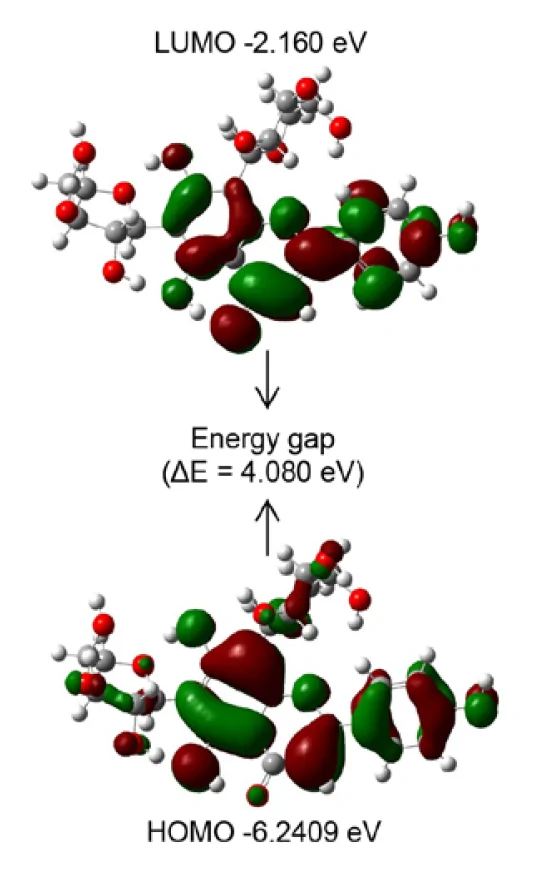
Justridusamide A	6.0711	0.8775	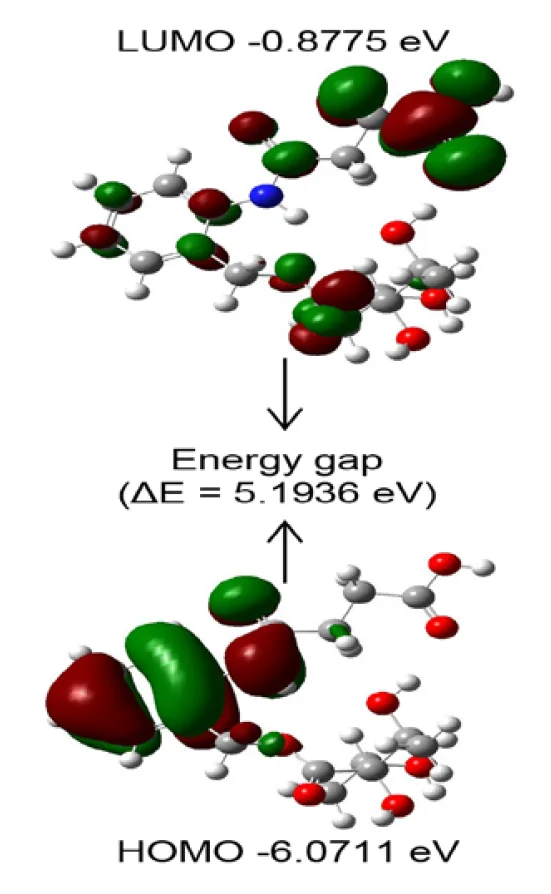
Methyl 2-[cyclohex-2-en-1-yl(hydroxy) methyl]-3-hydroxy-4-(2-hydroxyethyl)-3-methyl-5-oxoprolinate	6.3427	0.3695	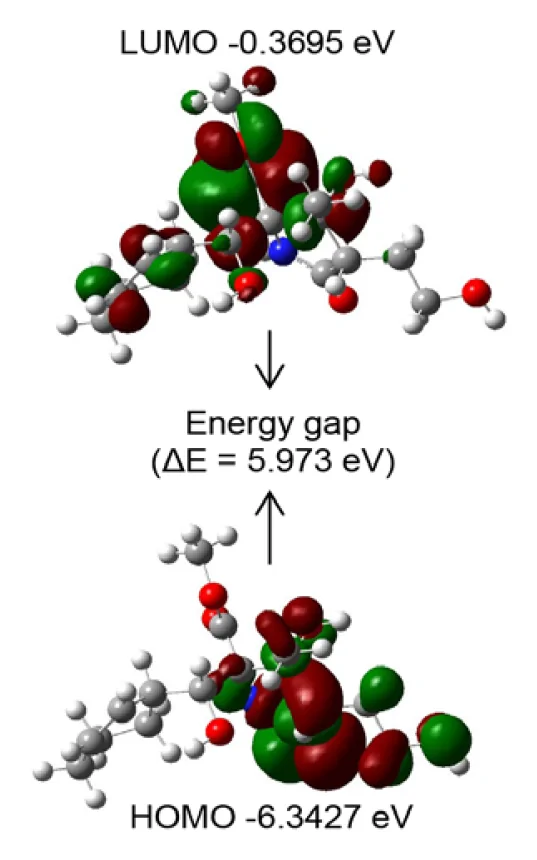
Sesamol	5.5508	0.124	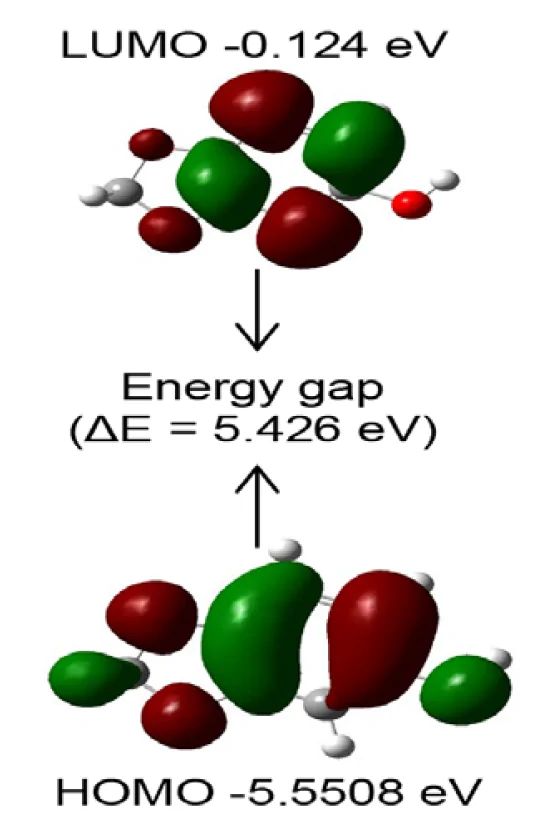
Trolox	5.2139	0.4481